# Supplementary material for: Chlorantraniliprole Enhances Cellular Immunity in Larvae of Spodoptera frugiperda (Smith) (Lepidoptera: Noctuidae)
Source: Insects. 2024 Aug 1;15(8):586. doi: 10.3390/insects15080586 (PMC11354488; doi:10.3390/insects15080586)
Supplement: Supplementary file 1 [file insects-15-00586-s001.zip › insects-3095374-supplementary.pdf]

Table S1. Composition of artificial diet for rearing *Spodoptera frugiperd*a larvae.

**Components**	**Prescription**
Maize powder	450 g
Soybean powder	150 g
Yeast powder	150 g
Casein	12.5 g
Agar powder	35 g
Multi-vitamins B	0.2 g
Roxithromycin	0.15 g
Ascorbic Acid	12.5 g
Sorbic Acid	2.25 g
Anhydrous Citric Acid	7.5 g
Methyl 4-Hydroxybenzoate	5 g
Choline chloride	2.25 g
Distilled Water	2 L
